# An Explainable Machine Learning Approach for COVID-19’s Impact on Mood States of Children and Adolescents during the First Lockdown in Greece

**DOI:** 10.3390/healthcare10010149

**Published:** 2022-01-13

**Authors:** Charis Ntakolia, Dimitrios Priftis, Mariana Charakopoulou-Travlou, Ioanna Rannou, Konstantina Magklara, Ioanna Giannopoulou, Konstantinos Kotsis, Aspasia Serdari, Emmanouil Tsalamanios, Aliki Grigoriadou, Konstantina Ladopoulou, Iouliani Koullourou, Neda Sadeghi, Georgia O’Callaghan, Eleni Lazaratou

**Affiliations:** 1 University Mental Health Research Institute, 11527 Athens, Greece; icedale@gmail.com (D.P.); mariana.har.travlos@gmail.com (M.C.-T.); ioannarannou@gmail.com (I.R.); 2 First Psychiatric Department, Eginition Hospital, National and Kapodistrian University of Athens, 11528 Athens, Greece; nadia.magklara@gmail.com (K.M.); elazar@med.uoa.gr (E.L.); 3 Second Psychiatric Department, ‘Attikon’ University Hospital, National and Kapodistrian University of Athens, 12462 Athens, Greece; igioannag@gmail.com; 4 Department of Psychiatry, Faculty of Medicine, School of Health Sciences, University of Ioannina, 45110 Ioannina, Greece; konkotsis@gmail.com; 5 Department of Child and Adolescent Psychiatry, Medical School, Democritus University of Thrace, University Hospital of Alexandroupolis, 68100 Alexandroupolis, Greece; aserntar@med.duth.gr; 6 Department of Child and Adolescent Psychiatry, Division of Psychiatry, ‘Asklepieion Voulas’ General Hospital, 16673 Attica, Greece; emtsalamanios@hotmail.com; 7 Hellenic Centre for Mental Health and Research, 10683 Athens, Greece; alikigrigoriadou@gmail.com; 8 Athens Child and Adolescent Mental Health Centre, General Children’s Hospital ‘Pan. & Aglaia Kyriakou’, 11527 Athens, Greece; kladopou@gmail.com; 9 Mental Health Center, General Hospital ‘G. Hatzikosta’, 45445 Ioannina, Greece; jkoullourou@gmail.com; 10 Section of Clinical and Computational Psychiatry, National Institute of Mental Health, National Institutes of Health, Bethesda, MD 20892, USA; neda.sadeghi@nih.gov (N.S.); georgiaocallaghan@gmail.com (G.O.)

**Keywords:** COVID-19 pandemic, children and adolescents, machine learning, post hoc explainability, model calibration

## Abstract

The global spread of COVID-19 led the World Health Organization to declare a pandemic on 11 March 2020. To decelerate this spread, countries have taken strict measures that have affected the lifestyles and economies. Various studies have focused on the identification of COVID-19’s impact on the mental health of children and adolescents via traditional statistical approaches. However, a machine learning methodology must be developed to explain the main factors that contribute to the changes in the mood state of children and adolescents during the first lockdown. Therefore, in this study an explainable machine learning pipeline is presented focusing on children and adolescents in Greece, where a strict lockdown was imposed. The target group consists of children and adolescents, recruited from children and adolescent mental health services, who present mental health problems diagnosed before the pandemic. The proposed methodology imposes: (i) data collection via questionnaires; (ii) a clustering process to identify the groups of subjects with amelioration, deterioration and stability to their mood state; (iii) a feature selection process to identify the most informative features that contribute to mood state prediction; (iv) a decision-making process based on an experimental evaluation among classifiers; (v) calibration of the best-performing model; and (vi) a post hoc interpretation of the features’ impact on the best-performing model. The results showed that a blend of heterogeneous features from almost all feature categories is necessary to increase our understanding regarding the effect of the COVID-19 pandemic on the mood state of children and adolescents.

## 1. Introduction

In December 2019 the World Health Organization (WHO) identified the novel coronavirus (COVID-19) as the cause of pneumonia in Wuhan, China, and on 11 March 2020 the WHO declared COVID-19 as a pandemic [1,2]. Between 31 December 2019 and 4 May 2020, over 184 countries adopted strict measures to limit the spread of COVID-19, such as lockdown restrictions and quarantine time, which led to socioeconomic, environmental, and mental health challenges. Within those restrictions, specific measures ranged from working from home, to online education (e-learning), to social restrictions to border closures (Table 1) [3]. Even though the lockdown policies contributed to the control and decrease in the spread of COVID-19, they also resulted in the deterioration of the mental health of the population worldwide [3,4,5]. 

A plethora of studies have been conducted to examine the impact of COVID-19 and its restriction policies on the studied population [6,7,8]. Specifically, multivariable logistic regression analyses were adopted in various studies to: (i) identify the correlations of mental health with other factors [9,10,11], such as sociodemographic features [4,12,13,14] and/or school aspects [14] or health behaviors [15], mostly on university students [16,17,18]; (ii) assess the prevalence and the risk factors associated with self-reported psychological distress [19]; and (iii) evaluate the effects of COVID-19 measures upon the mental health of children and adolescents, with or without pre-existing diagnoses [20]. Binomial or binary logistic regression analysis was used to: (i) identify sleeping problems of adolescents and young adults (12–29 years) during the pandemic [21,22]; (ii) assess depression and anxiety amongst university students [23]; and (iii) examine the prevalence of anxiety among children and the possible association to COVID-19 [24]. Other studies focused on youths used univariate logistic regression to identify mental health issues [25]. Hierarchical logistic regression analyses were used to examine variables associated with mental health problems during the COVID-19 outbreak to university students [26]. Adjusted logistic regression analyses were used to examine the association between stress due to COVID-19 and worries to children and adolescents [27]. However, limited studies have been employed with machine learning prediction models such as the XGBoost model, to predict anxiety and insomnia in undergraduate students during the COVID-19 pandemic [28], or random forest and regression trees to identify predictors of psychological distress during COVID-19 in participants aged 18–85 [29].

Most of the above presented studies focused on Chinese regions [14,16,26] and college students [16,19,26], and used traditional statistical approaches such as logistic regression and chi-square tests [23,24,25,27] to identify correlations among risk factors and mental health problems, while only few of them employ machine learning methodologies [29]. Furthermore, to the best our knowledge, there has not been any study focused on children and adolescents with diagnosed mental disorders. Therefore, this study aims to fill this gap by proposing the development of an explainable machine learning pipeline to create a deeper understanding of the consequences and impact of the first lockdown in Greece on the mental health of children and adolescents. The study includes 71 heterogenous factors. The proposed methodology consists of: (i) clustering the examined population based on their mood state alteration during lockdown; (ii) identifying the main features that contribute to the mood alteration of the examined population; (iii) developing calibrated machine learning models to predict the alteration of mood state; (iv) post hoc explainability analysis to rank features in terms of their impact on the final machine learning outputs. 

The current study focuses on children and adolescents that had been attending Children and Adolescents Mental Health Services (CAMHS) in Greece during the year prior to the pandemic. 

## 2. Background

Recent studies have focused on a statistical or machine learning approach to predict or interpret the impact of COVID-19 on the mental health of children and adolescents. Regarding participants, only a limited number of studies have focused on children and young adults (Table 2). Specifically, a multivariable logistic regression analysis was performed in order to identify correlations between sociodemographic features and mental health problems in Chinese adolescents during the outbreak of COVID-19. The population was composed of 8079 Chinese students aged 12–18. The data were collected by the Patient Health Questionnaire (PHQ-9) and the Generalized Anxiety Disorder (GAD-7) questionnaire with the goal of assessing depressive and anxiety symptoms. Results showed that female students and those with higher grades had an elevated risk of presenting symptoms of anxiety and depression [2]. Moreover, a second survey was conducted with regards to the mental health of Chinese children aged 7–15 years during COVID-19, with a total of 668 parents across different regions of China. Multiple logistic regression analysis was used to analyze the data, identifying the main factors that contribute to the education and the mental health of Chinese children, and found the school system and province of origin to be significant factors associated with developing PTSD, and the majority of participants having a positive opinion about online education [4]. Liang et al. studied the effects of COVID-19 on youth mental health in China by collecting data from the General Health Questionnaire (GHQ-12), the PTSD Checklist—Civilian Version (PCL-C) and the Negative coping styles scale from 584 youths. The univariate analysis and univariate logistic regression showed that almost 40.4% of the sampled youth were found to be prone to psychological problems, and 14.4% to post-traumatic stress disorder (PTSD) symptoms [25].

A comparison among two cross-sectional studies was conducted to evaluate the factors that contributed to depression and anxiety among Chinese adolescents during the COVID-19 pandemic [14]. The first study took place between 20 February and 27 February, while the second between 11 April and 19 April 2020; The studies had 9554 and 3886 participants, respectively. Multivariable logistic regression analyses revealed that group membership in the second survey, female gender, senior secondary school enrollment, and concerns about entering a higher grade were positively associated with both depression and anxiety [14]. 

Another study assessed prevalence and risk factors associated with self-reported psychological distress amongst 1,199,320 school-aged children and adolescents in China, between 8 March and 30 March 2020. Multivariate logistic regression and odds ratio showed that 126,355 students reported psychological distress, and that older children had an increased risk of experiencing psychological distress, as did students who never wore face masks and those who spent less than 0.5 h exercising [19]. Another online survey focusing on 11,835 Chinese adolescents and young adults (12–29 years) was conducted regarding sleeping problems during the pandemic [21]. The Pittsburgh Sleep Quality Index (PSQI), the PHQ-9, and GAD-7 questionnaires were used to assess insomnia, depression, and anxiety symptoms, respectively, while the Social Support Rate Scale was used to assess social support. Binomial logistic regression analysis revealed that high risk factors for presenting insomnia symptoms were being female and residing in the city [21]. 

Most of the studies have focused on college students. Ge et al. used the XGBoost model to predict anxiety and insomnia in Chinese undergraduate students during the COVID-19 pandemic. In total, 2009 students participated by answering questionnaires during the two first moths attending university, between 10 and 13 February 2020. The results showed that the most related variables in predicting anxiety included romantic relationships, suicidal ideation, sleep problems, and a history of anxiety symptoms, while the prediction of insomnia was found to be associated with aggression, psychotic episodes, suicidal ideation, and romantic relationships [28]. Another study focused on 746,217 Chinese university students, which conducted univariate and hierarchical logistic regression analyses to examine variables associated with mental health problems during the COVID-19 outbreak in 2019. Results showed that being in close relation to others who had contracted the virus, exposure to social media coverage of COVID-19 for more than three hours daily, and inadequate social support were the main contributing factors to mental health problems among participants [26]. Additionally, a study of 89,588 Chinese university students found that 36,865 students reported anxiety symptoms, and multivariate logistic regression models showed that risk factors for anxiety symptoms included being 26–30 years old, being in sophomore, junior and senior grades, having a higher paternal education level, low economic status, or low social support [16]. Among 933 medical students who participated in a cross-sectional survey evaluating the impact of COVID-19 between 4 and 12 February 2020 and completed the PHQ-9 and GAD-7, anxiety was found in 17.1% of participants and depression in 25.3% of participants. Furthermore, anxiety levels were higher among those located in the Wuhan epicenter, rather than Beijing [17]. 

Several studies have also focused on French university students. A study with a total of 69,054 participants who completed a survey between 17 April and 4 May 2020 showed a high prevalence of mental health issues among students who experienced quarantine, which highlighted the need for prevention, surveillance, and access to care [18]. Another study with a sample of 3671 participants who completed an online retrospective survey between the 13 March and 11 May 2020 found a significant reduction in tobacco smoking, binge drinking, and cannabis use, while reductions in physical activity were associated with higher depression levels and being male [15]. 

A web-based cross-sectional survey assessed depression and anxiety amongst 476 university students during the COVID-19 pandemic in Bangladesh, using binary logistic regression. Results showed that older students were more likely to have greater depression, whereas students who afforded private tuition during the pre-pandemic period had depression [22]. Furthermore, an online cross-sectional study conducted in Bangladesh between 15 April and 9 May, gathered data from 384 parents with at least one child aged 5–15 [23]. Results indicated that 43% of children rated over the subthreshold on mental disturbances, 30.5% mild disturbances, 19.3% moderate disturbances, and 7.2% severe disturbances. Lastly, higher percentages of mental health disturbances were associated with higher parental education levels, parents attending to the workplace, and relatives infected with COVID-19 [23].

Cost et al. (2021) [20] evaluated the effects upon the mental health of children and adolescents, with or without pre-existing diagnoses, in response to the emergency measures set in place for COVID-19 in Canada. For parents of children aged 6–18, the Coronavirus Health and Impact Survey (CRISIS) questionnaire, along with self-reports, was used in order to examine mental and behavioral changes, while for children aged 2–5, the Strengths and Difficulties Questionnaire (SDQ) was used. Multinomial logistic regression identified that during the first wave of the pandemic there was a deterioration in the mental health of children and adolescents with and without previous diagnosis, with the former experiencing greater deterioration and greater stress related to social isolation. For some children, the impact of a pre-existing diagnosis was associated with deterioration in depression, irritability, hyperactivity, and obsessions/compulsions, while for others it was associated with an improvement in anxiety, attention, and obsessions/compulsions. 

An additional study examined the prevalence of anxiety among Brazilian children, and the possible association to COVID-19, during April and May 2020 [24]. 157 girls and 132 boys aged 6–12, along with their parents or guardians, participated in the study. Using the Children’s Anxiety Questionnaire (CAQ) and the Numerical Rating Scale (NRS), data showed that children whose parents had essential jobs and were social distancing had higher levels of anxiety, while results from the logistic regression suggested that social distancing without parents, a higher number of people per household, and the education level of parents or guardians, were also associated with higher anxiety scores in CAQ.

Tamarit et al. (2020) examined the association between sociodemographic factors and COVID-19-related variables and their effect on depression, anxiety, and stress among adolescents in Spain [13]. A total of 523 adolescents (13–17 years) completed the Depression, Anxiety and Stress Scale (DASS-21) along with the Oviedo Infrequency Scale (INFO-OV), with results indicating that girls who work voluntarily and those who stayed home more frequently were more likely to show symptoms of depression, anxiety, or stress. In addition, the study indicated an association between mental distress and stressful life events whilst conducting research related to COVID-19. Finally, participants who were in a romantic relationship, along with those who had already been infected with COVID-19, were more likely to have an improved mental health state.

In addition to the above, a study focused on children and adolescents aged 5–17 with attention deficit hyperactivity disorder (ADHD) aimed to identify the impact of COVID-19 restrictions in Australia [27]. Parents of 213 children who had been diagnosed with ADHD participated on the survey in May 2020, during COVID-19 restrictions. The study focused on: (i) child physical health, media use, and mental health; (ii) life changes; (iii) changes and/or barriers to healthcare, among others. Statistical analysis indicated that COVID-19 restrictions were associated with decreased exercise, outdoor time, and enjoyment in activities, and an increase in watching television, social media use, and gaming, as well as increases in depressed mood and loneliness. On the contrary, 64% of parents identified increased family time and positive changes. 

Another cross-sectional study based on machine learning examined the psychological impact of COVID-19 on 478 college students after school reopening [19]. Results indicated that students who experienced fear of being infected, a pessimistic attitude, friends of family contracting COVID-19, and higher grades easily experienced anxiety or depression. Multivariate logistic regression indicated a variety of significant factors influencing anxiety or depression, including alcohol use, school reopening, taking temperature routinely, sleep quality, lockdown restrictions, and availability of package deliveries.

A Belgian survey examined mental distress and its contributing factors among 2008 young people aged 16–25 years during the first wave of COVID-19, using Bivariate and multivariable logistic regression analyses. The results showed that approximately two-thirds of the participants experienced mental distress. They also found that low social support, loneliness, social media use, decreased participation in social situations, being female, and decreased completion of home activities to be significant predictors of mental distress [31]. Another study focused on identifying predictors of psychological distress during COVID-19 in 2787 participants aged 18–85. Random forest machine learning algorithm and regression trees suggest that female participants, participants with underlying medical conditions, and those with emotional-based coping experienced higher levels of severe anxiety [29]. Finally, another cross-sectional study examined the mental health of 280 school-aged children in Florida, during the first COVID-19 long-distance-learning mandates. Bivariate analysis and logistic and multinomial logistic regression models showed that loss of household income and being female were associated with being at higher risk for anxiety symptoms, depressive symptoms, and OCD symptoms, whereas parental protective practices against COVID-19 were found to increase the risk of depressive symptoms [10]. 

Most of the above presented studies focused on Chinese regions [14,16,26] and college students [16,19,26], and used traditional statistical approaches, such as logistic regression and chi-square tests [23,24,25,27] to identify correlations among risk factors and mental health problems, while only few of them employed machine learning methodologies [29]. Furthermore, to the best our knowledge, there has not been any study focused on children and adolescents with diagnosed mental disorders, apart from a study focused on specific diagnosis [27]. Therefore, the contribution of our study is summarized as:

- The use of an explainable machine learning pipeline with multiple comparative evaluations among the ML stages to guarantee the development of an accurate prediction model;
- The use of a post hoc explainability model to diagnose and interpret the most contributed factors to the prediction output of the model and thus to identify the factors that led to mood alteration or stability during the first lockdown in Greece;
- The incorporation of 71 heterogeneous features from 10 different categories, such as demographics, social life, personal life, family life, daily activities, health concerns and behavioral effects, sleep habits, mood state, and medical diagnosis/rehabilitation;
- The application to the vulnerable group of population [31], such as children and adolescents with pre-existing psychiatric and/or developmental disorders, is incorporated in order to further understand the impact of COVID-19 and its restrictions by identifying the factors that contributed most to the mood state alteration of the population under examination during the first lockdown in Greece. To achieve this, machine learning tools were employed following a post-hoc explainability analysis.

## 3. Materials and Methods

**Figure 1:**
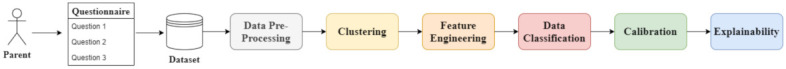
Machine learning pipeline adopted in this study.

To predict the impact of COVID-19 due to the first lockdown imposed in Greece during the period from 23 March 2020 to 4 May 2020, we focused on the sensitive group of children and adolescents. The data from the Hellenic COVID-19 imPact survEy (HOPE) were used, a longitudinal study surveying parents of children that had been attending, during the year prior to the pandemic (1 March 2019 to 1 March 2020), CAMHS in Greece (seven in Athens Greater Metropolitan Area, two in Ioannina, one in Alexandroupolis, one in Thessaloniki, and one on Crete). A machine learning pipeline (Figure 1) was proposed that included: (i) data collection via questionnaires and medical reports; (ii) data preprocessing; (iii) a competitive evaluation of state-of-the art clustering methods and evaluation metrics; (iv) a feature selection based on a state-of-the-art and robust method, named ReliefF, that has been proven effective for medical data; (v) a competitive evaluation of various ML models following calibration; and (vi) a post hoc explainability of the best performed model with SHAP to identify the features’ impact on the model.

### 3.1. Data Collection

To collect the data and form the dataset, children who attended the service of CAMHS participated. Specifically, 744 children whose parents (738 parents) answered the online questionnaire on their behalf participated in this study. This process took place between 8 May and 1 June 2020. The questionnaire included questions relevant to demographic information, parent’s evaluation of the child’s condition 3 months (3m) before the lockdown and 2 weeks (2w) after the first lockdown in Greece. Table 3 shows the sociodemographic characteristics of the dataset, while Table 4 presents the description of the variables used in the study as they were extracted from the questionnaires. 

### 3.2. Data Preprocessing

Data imputation was not needed since there were no missing values of categorical or numerical variables in the final dataset. Furthermore, as a common requirement for many ML classifiers, the standardization of the dataset was implemented. 

### 3.3. Clustering Methods

**Figure 2:**
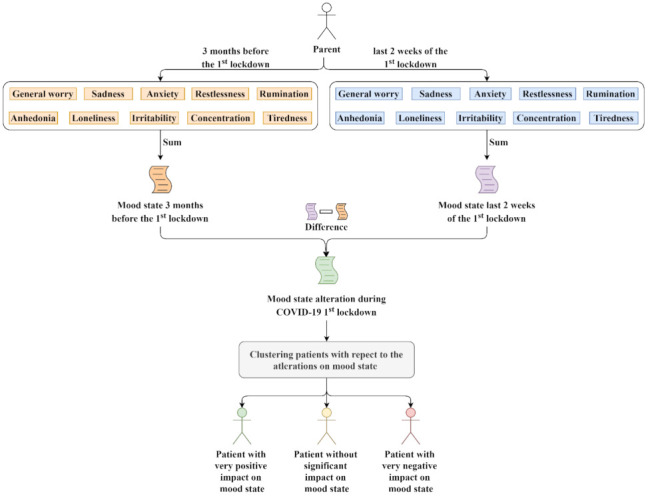
Clustering process.

For the clustering process, six popular methods were employed, such as Mini Batch K-Means [32], Spectral Clustering [33], Ward [34,35], Average Linkage [36,37], Balanced Iterative Reducing and Clustering using Hierarchies (Birch) [38,39], and Jenks natural breaks optimization method (Jenks) [40,41,42]. Clustering was performed on the values of the variable mood_change, that represents the change in mood state (Figure 2). Specifically, the mood state score prior to the lockdown (Equation (1)) and during the lockdown (Equation (2)) is calculated by the sum of the variables general_worry, sadness, anxiety, restlessness, anhedonia, loneliness, irritability, concentration, tiredness, and rumination (Table 4). The change in mood state is the difference between their mood state score during the last 2 weeks and 3 months before the first lockdown in Greece (Equation (3)). Hence, a negative value of the predicted variable mood_change indicates an overall improvement of the subject’s mood state score, while a positive value indicates an overall worsening of the subject’s mood state score. Values close to zero show that there was no change in the subject’s mood state score during the lockdown.

(1) $$\begin{array}{c} 3m\_\text{mood}\_\text{state}\;=\;3m\_\text{general}\_\text{worry}\;+\;3m\_\text{sadness}\;+\;3m\_\text{anxiety}\;+\\ 3m\_\text{restlessness}\;+\;3m\_\text{anhedonia}\;+\;3m\_\text{loneliness}\;+\;3m\_\text{irritability}\;+\\ 3m\_\text{concentration}\;+\;3m\_\text{tiredness}\;+\;3m\_\text{rumination} \end{array}$$

(2) $$\begin{array}{c} 2w\_\text{mood}\_\text{state}\;=\;2w\_\text{general}\_\text{worry}\;+\;2w\_\text{sadness}\;+\;2w\_\text{anxiety}\;+\\ 2w\_\text{restlessness}\;+\;2w\_\text{anhedonia}\;+\;2w\_\text{loneliness}\;+\;2w\_\text{irritability}\;+\\ 2w\_\text{concentration}\;+\;2w\_\text{tiredness}\;+\;2w\_\text{rumination} \end{array}$$

(3) mood_change = 2w_mood_state − 3m_mood_state

### 3.4. Feature Engineering

The feature selection process was performed by using the ReliefF algorithm, due to its effectiveness in medical diagnosis and medical classification problems [43,44,45,46,47]. ReliefF is an extension of the original Relief which can deal with multiclass problems due to its enhancement with noise resistance [48,49], and therefore it is considered suitable for the current medical multiclass classification problem, as defined in Section 3.3, Figure 2. 

### 3.5. Data Classification

To solve the defined multiclass classification problem, seven popular classifiers (Table 5) are employed and tested: Random Forest (RF), Multi-Layer Perceptron (MLP), Extreme Gradient Boosting (XG Boost), Logistic Regression (LR), Support Vector Machine (SVM), K-Nearest Neighbor (KNN), and Decision Trees (DT). The adopted models are frequently used for medical classification problems while covering various types of prediction models such as tree-based, linear, or neural networks [50,51,52,53,54,55].

### 3.6. Post Hoc Explainability

In the current study, the Shapley Additive exPlanations (SHAP) is employed to rank the features of the dataset with respect to their impact on the final machine learning outputs. SHAP calculates optimal Shapley values from coalitional game theory. These values show how fairly the impact on a model’s prediction is distributed among the features of the dataset. Then, SHAP develops a mini-explainer model that corresponds to a single-row-prediction pair in order to explain how this prediction was achieved [62]. 

## 4. Results

### 4.1. Evaluation Methodology

**Figure 3:**
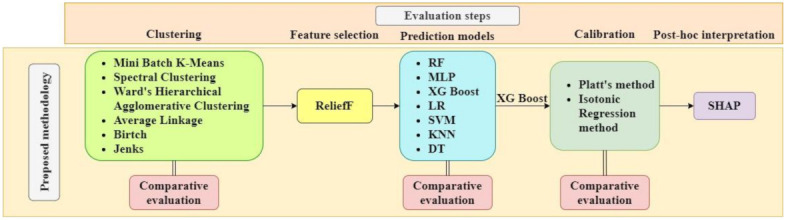
Evaluation methodology.

The proposed methodology was applied in the context of predicting the change in the mood state of children and youths that are diagnosed with a mental disease, by using the medical data derived from the dataset (Section 3.1). Initially, an evaluation of the best-performed clustering method is performed; then, based on the results of the feature selection method, various prediction models are evaluated to choose the best-performed based on the accuracy metric following a calibration process. For the best-performing calibrated model, a post hoc explainability analysis is performed for a deeper understanding and interpretation of the most contributing features to the model’s output (Figure 3). 

**Figure 4:**
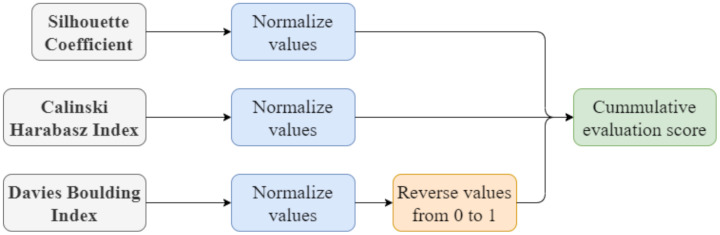
Evaluation process of clustering methods.

The three clustering evaluation criteria that are combined are the Silhouette Coefficient, the Calinski–Harabasz Index, and the Davies–Bouldin Index. Specifically, the normalized scores of the evaluation criteria are summed for calculating a cumulative evaluation score (Figure 4). The default parameter settings from sklearn.cluster module (https://scikit-learn.org/stable/modules/classes.html#module-sklearn.cluster, accessed on 1 August 2021) were used for the clustering methods, while the additional settings are shown in Table 6 Then, the feature selection is performed with ReliefF on the three clusters derived by the prevailing clustering method (Figure 4). For the classification, a repeated stratified 5-fold cross validation with grid search was adopted with SMOTE method [63,64]—oversampling to training dataset for the minority classes. The prediction models were evaluated in subsets of features with increasing dimensionality. The accuracy was chosen as the evaluation criterion for the performance of the prediction models. Table 7 presents the hyperparameters of the classification models for tuning. 

### 4.2. Results

#### 4.2.1. Clustering 

Table 8 shows the results from the clustering methods that were employed to group the population among the individuals with positive change to their mood state (Cluster 0), without significant change (Cluster 1) and with negative change (Cluster 2). Table 9 shows the evaluation score achieved by each clustering method. 

#### 4.2.2. Feature Selection

**Figure 5:**
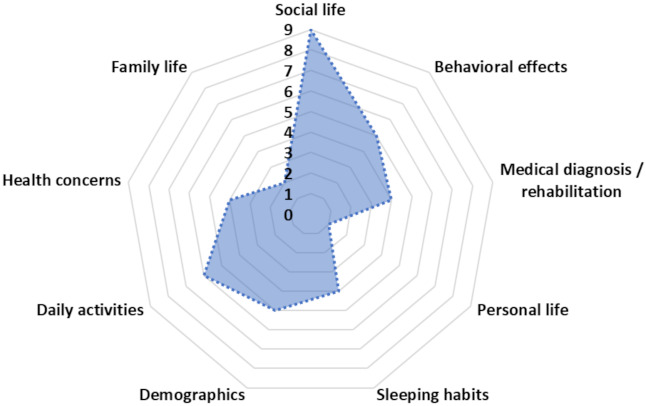
Spider plot of the number of features that belong to each feature category for the first 40 features where the best performance was achieved.

Table 10 shows the 40 most significant features of our dataset derived from ReliefF, while Figure 5 illustrates the spider plot with the number of features from each category for the first 40 features where the best performance was achieved.

#### 4.2.3. Classification and Calibration

**Figure 6:**
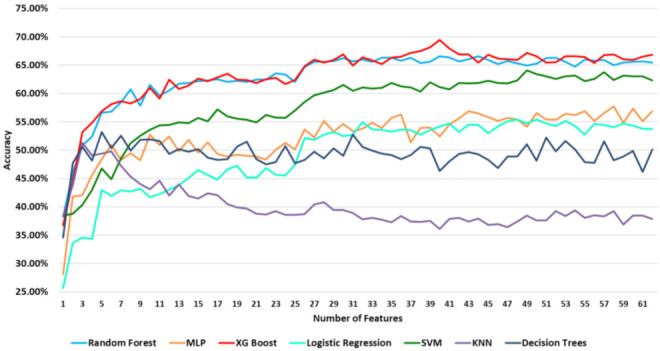
Classification results.

Figure 6 illustrates the accuracy of the comparative prediction models per number of features. Table 11 shows the maximum achieved accuracy of each prediction model used in the experimental evaluation and the number of features where the maximum accuracy was reached. 

**Figure 7:**
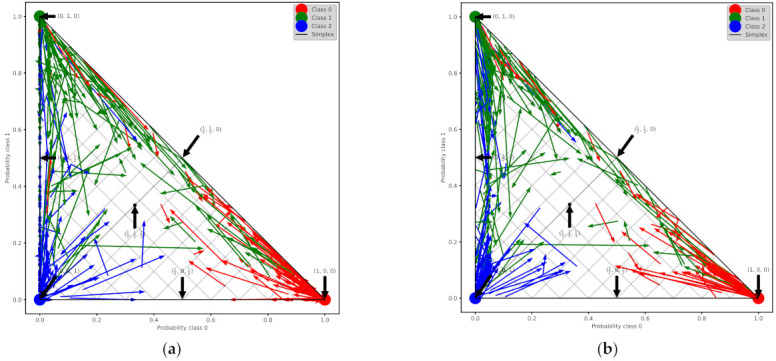
Change of predicted probabilities on test samples after calibration with: (**a**) Isotonic Regression method; (**b**) Platt’s (sigmoid) method.

**Figure 8:**
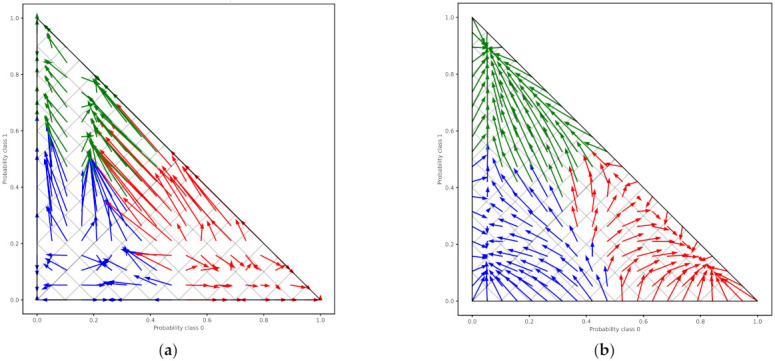
Learned calibration map with: (**a**) Isotonic Regression method; (**b**) Platt’s (sigmoid) method.

**Figure 9:**
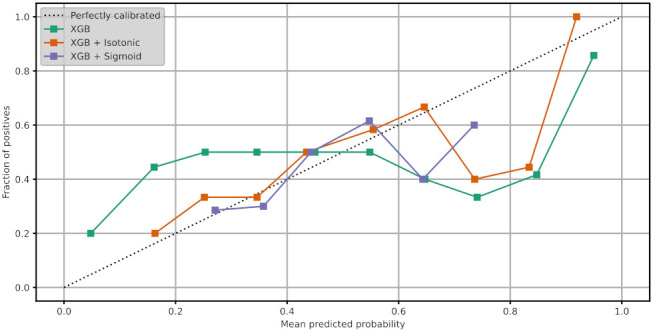
Calibration plot of XG Boost classifier for class 0.

**Figure 10:**
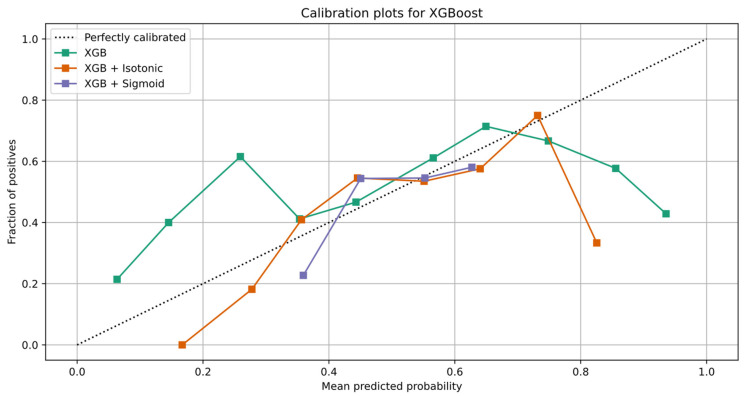
Calibration plot of XG Boost classifier for class 1.

**Figure 11:**
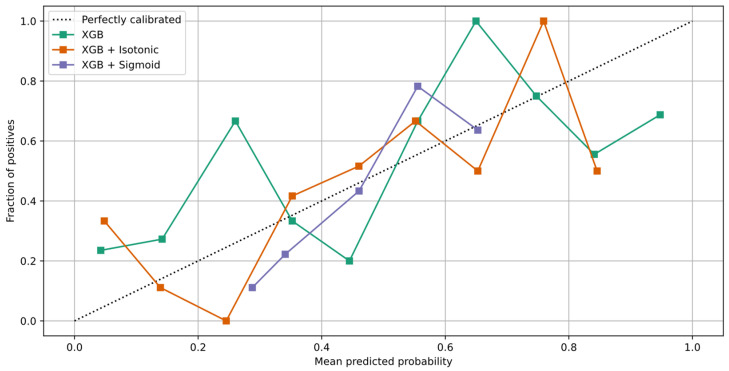
Calibration plot of XG Boost classifier for class 2.

To increase the performance of the XG Boost model, we perform calibration with Isotonic Regression and Platt’s methods. We use the logistic regression loss (Log-loss) and the accuracy to evaluate the models. Table 12 shows the results after XG Boost classifier calibration with Isotonic Regression and Platt’s methods. Figure 7a,b depicts the change of predicted probabilities on test samples after calibration with Isotonic Regression and Platt’s (sigmoid) methods, respectively. The red, green, and blue colors of an arrow represent the true classes 0, 1, and 2, respectively. Class 0, class 1, and class 2 represent the patients with negative, neutral, and positive change on their mood state, respectively. Figure 8a,b depicts the learned calibration maps. The learned calibration map consists of a grid of possible uncalibrated probabilities over the 2-simplex by computing the corresponding calibrated probabilities and plot arrows for each. The arrows are colored according to the highest uncalibrated probability. Figure 9, Figure 10 and Figure 11 illustrate the calibration plots for each class over the others.

#### 4.2.4. Post-Hoc Explainability 

**Figure 12:**
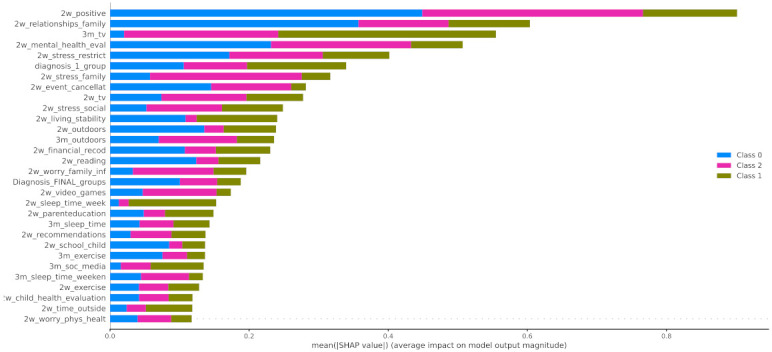
Mean SHAP values.

**Figure 13:**
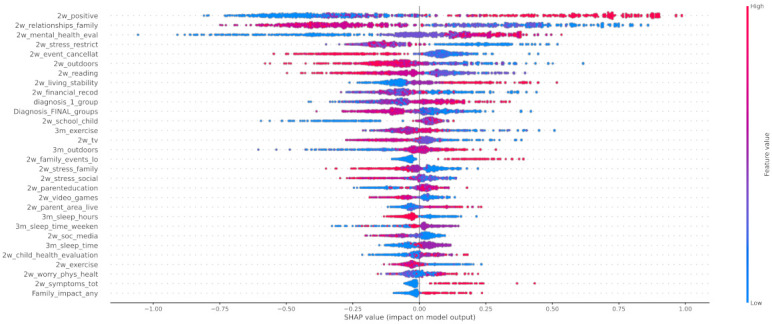
SHAP values of patients from class 0.

**Figure 14:**
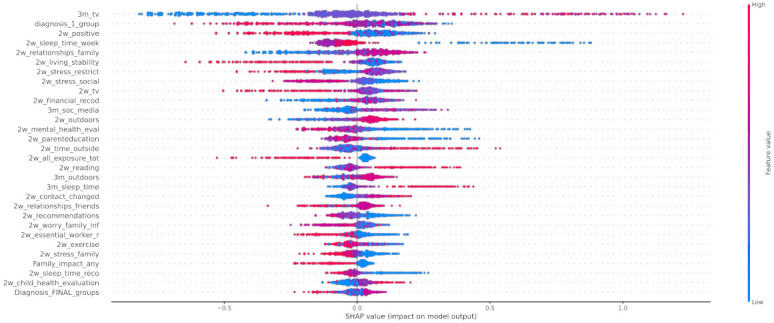
SHAP values of patients from class 1.

**Figure 15:**
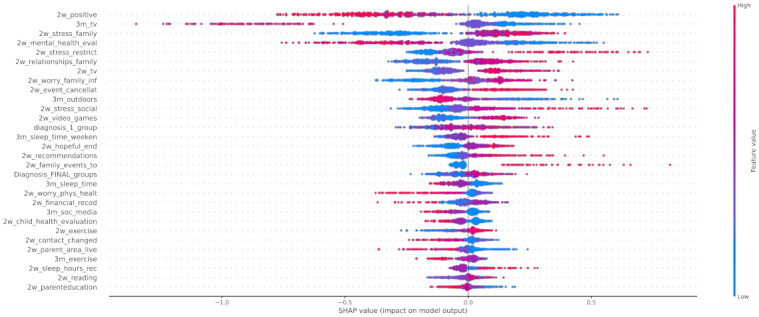
SHAP values of patients from class 2.

**Figure 16:**
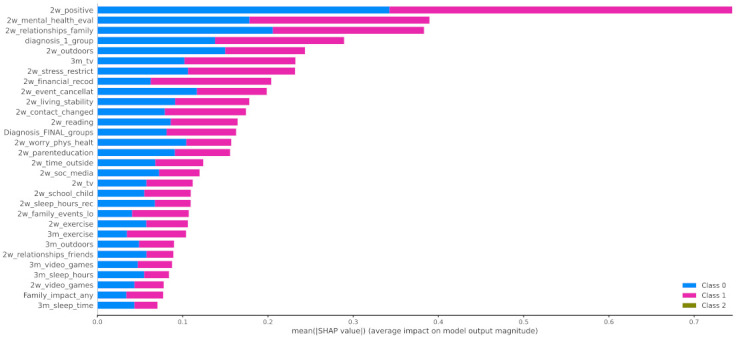
Mean SHAP values of patients from class 0 and class 1.

**Figure 17:**
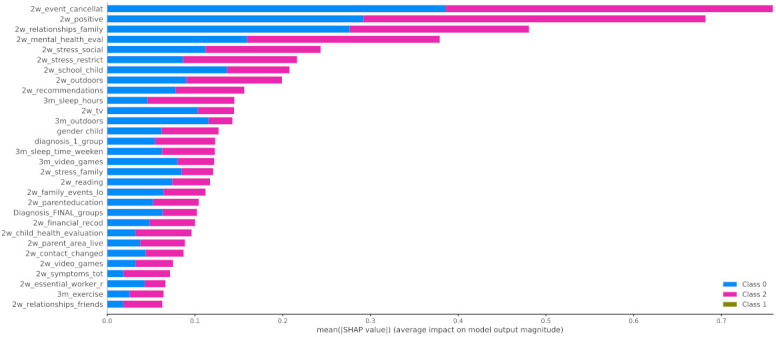
Mean SHAP values of patients from class 0 and class 2.

**Figure 18:**
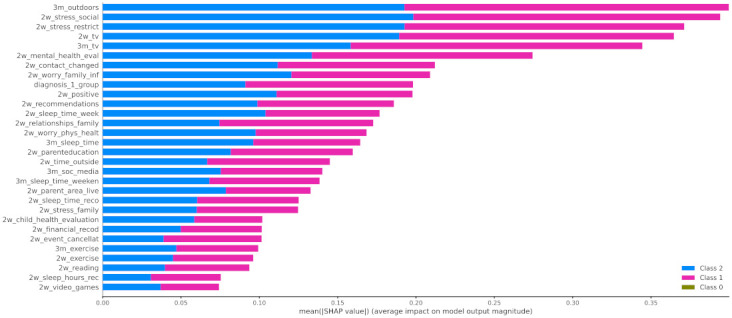
SHAP values patients from class 1 and class 2.

In Figure 12 the x-axis represents the average magnitude change in model output when a feature is excluded from the model. The higher the value, the higher the importance of this feature in the prediction outcome of the model. In Figure 13, Figure 14 and Figure 15, the feature names are presented in *y*-axis based on their importance from top to bottom, while the *x*-axis indicates the mean SHAP value showing the change in log-odds. Gradient color (red to blue) indicates the original value of that feature. Each point represents a patient from the original dataset. Figure 16, Figure 17 and Figure 18 show the mean SHAP values of each feature that affects the classification of a patient between two groups. 

## 5. Discussion

### 5.1. Clustering

The clustering results indicated that the Jenks method is the most suitable to be adopted in our study, reaching the highest evaluation score (Table 9). The clusters derived from the Jenks method indicate that most of the individuals that participated in this study (469 out of 744, 63.04%) did not have any significant alteration to their mood state (Table 8). Also, it is important to mention that the first lockdown in Greece had a negative impact on more individuals (169, 22.71%) than it had positive (106, 14.25%).

### 5.2. Feature Selection

The results revealed that social life aspects play a significant role in the prediction output (Table 10, Figure 5). Indeed, the spider plot, depicted in Figure 5, reveals that nine features from the social life category appeared in the 40 most significant features. Furthermore, daily activities is the second most important category, with six features in the feature selection subset. Finally, behavioral effects and demographics contribute with five features each. The remaining features belong to the categories of medical diagnosis/rehabilitation, sleeping habits, health concerns, family life, and personal life (Figure 5). The above results clearly indicate that features from all categories are needed to accurately predict the impact of COVID-19 on the mood states of children and adolescents.

### 5.3. Classification and Calibration

The results in Table 11 showed that the XG Boost model presented a more stable performance compared to the other models, achieving the maximum accuracy (69.47%) at 40 features. A comparable performance (66.60%) was also achieved by Random Forest at 44 features.

The calibration results showed that the calibrated XG Boost with Isotonic Regression achieved lower log-loss but also slightly lower accuracy compared to the calibrated XG Boost with Platt’s method (Table 10). In Figure 7a,b the vertexes of the simplex represent the perfectly predicted classes (e.g., 0, 0, 1). The middle point $\left(\frac{1}{3},\frac{1}{3},\frac{1}{3}\right)$ inside the simplex represents the prediction of the three classes with equal probability $\left(\frac{1}{3},\frac{1}{3},\frac{1}{3}\right)$ . The start of an arrow is at the uncalibrated probabilities, while the head of an arrow shows the calibrated probability. For a lower overconfident model, the arrows point away from the edges where the probabilities of a class are zero. This can be better observed to the calibrated XG Boost with Platt’s method, which produces more accurately predicted probabilities, incurring a lower log-loss. 

The learned calibration maps showed that Platt’s method succeeded in calibrating the model better compared to the Isotonic Regression method. Indeed, this can also be observed in Figure 9, Figure 10 and Figure 11 where the calibration plots for each class over the others are illustrated. In all cases, the XG Boost model calibrated with Platt’s (sigmoid) method verges more to the perfectly calibrated line compared to the non-calibrated model or the XG Boost model calibrated with the Isotonic Regression method.

### 5.4. Post Hoc Explainability

In this study, the predicted variable was set to be the mood_change, i.e., the change in mood state before and during the first lockdown in Greece. The results showed that the change in the child’s mood state was highly associated with the parent’s perception on whether the COVID-19 crisis led to positive changes in their child’s life (2w_positive), their relationships among the family (2w_relationships_family) and the evaluation of their mental health before the COVID-19 crisis (3m_tv), as it is illustrated in Figure 12. In addition, an important contribution was proved to be the increase in the child’s time spent on watching TV or using digital means during the 3 months before and 2 weeks after the lockdown. Therefore, we can observe that there was a negative impact on children who did not use to spend much time watching TV but whose time increased due to lockdown. It is important to mention that the first diagnosis defined by a medical expert played a significant role in the change in the children’s mood state.

Regarding local exploration, Figure 15 shows that the most important features that contribute to classifying an individual to the group with negative change of mood state include the lack of positive changes to their life, the increase in watching tv, the stress derived from the restrictions, and the stress caused to the child by changes in family contacts. Regarding the individuals who had not been affected by the first lockdown imposed in Greece, the following features were found to contribute most to this category: 3m_tv, diagnosis_1_group, 2w_positive, and 2w_sleep_time_week. Based on Figure 14, responses indicate that a neutral attitude towards these features led to the classification of an individual as a child without mood state alteration. For instance, a child’s time spent watching TV was not affected significantly during the lockdown, but a more acceptable sleeping schedule for a child (sleeping time at 20:00–22:00) could lead to a more stable mood state. On the other hand, from the beeswarm in Figure 13, it is shown that more positive changes to their lives due to COVID-19, and better relationships with their family members, can lead to more positive behavior during the lockdown. Family cohesion and continuity in functional routines are protective factors that enhance mental resilience, involving a balance between adversity and availability of support. Protective factors act as a buffer against stress and moderate its impact on emotional well-being, as they enable children to cope with significant life events. Resilient family function provides children a sense of connectedness, healthy family attachments, and stability. Supportive parenting and family warmth facilitate stress exposure, and thus result in positive emotional development [65]. 

When it comes to the pairwise comparison among the groups, Figure 16 indicates that the main features that contributed to the distinction among the individuals who improved during the first lockdown and those whose mood state was not significantly affected were as follows: 2w_positive, 2w_mental_health_eval, and 2w_relationships_family. The most contributed features among the groups of children that had positive (class 0) or negative (class 2) changes to their mood state were 2w_event_canellat, 2w_positive, 2w_relationships_family, and 2w_mental_health_eval (Figure 17). Finally, the main features that contributed to the classification output among class 1 and class 2 were the 2w_event_canellat, 2w_positive, 2w_relationships_family, and 2w_mental_health_eval (Figure 18). 

Overall, we can conclude that if the first lockdown did not lead to positive changes, or negatively impacted the daily activities and family relationships of the child, then a deterioration in the mood state of a child was noticed. On the other hand, if COVID-19 restrictions did not affect the daily life and habits of the child (i.e., time spent watching TV, sleeping schedule), then no significant change to the mood state was noticed. Indeed, the stability on the functional routines constitutes a critical factor for the management of stressful events, such as a pandemic [66]. Finally, if during the first lockdown, children managed to change their life habits in a positive way, improved their relationships with family members, and were not affected by the cancellation of social events, then the change in their mood state was positive. Based on these conclusions, we can generalize that more outgoing and active children that did not use to spend more time at home watching TV prior to the pandemic were the most affected by the lockdown. On the other hand, children whose habits and daily life schedule did not alter significantly were the least affected by the COVID-19 restrictions. 

Apart from the features that have been included in the analysis, another perspective that should be considered and could probably explain the significant larger size of class 1 compared to the others (class 0 and 2) is the resilience in children and youth. Based on [67], resilience is defined as the capacity of a dynamic system to adapt successfully to challenges that threaten the function, survival, or development of the system. Various studies in the literature have highlighted the ability of children to adapt and benefit from their strengths and protective factors to succeed, despite biological and environmental influences, such as poverty, illness, violence, disasters, and family dissonance, among others [68,69,70], while few of them have focused on the case of COVID-19 [71]. Protective factors mainly include individual characteristics, environmental support, and family conditions. Indeed, in Figure 12, six factors are directly related to family conditions, such as relationships with family members, parental education, and financial stress, and nine factors are indirectly related to family and parental control, such as sleeping schedule and time dedicated to social media and TV. Moreover, nine factors are related to the ability of the child or youth to adapt to COVID-19 changes, such as changes to school attendance and social contacts, etc., while the remaining factors are linked with environmental supports, such as outdoor activities. 

## 6. Conclusions

In this study, an explainable machine learning pipeline was proposed to identify and interpret the most important features that contributed to the changes in the mood state of children and youths during the first lockdown in Greece. The aim of this study is to identify and understand, through the adopted ML pipeline, the factors that impacted the mental health of the examined population during the first COVID-19-related lockdown. Hence, to identify the changes in the mood state of the individuals under examination, the problem was formulated as a three-class classification problem. The classes included individuals with positive (class 0) and negative (class 2) changes in their mood state and individuals without a significant change in their mood state (class 1). A thorough comparative evaluation was conducted to identify the best-performed clustering method and prediction model for this problem. Jenks method was selected as the clustering method, following by a feature selection performed by ReliefF. The best-performed prediction model, XG Boost, was then used for calibration and a post hoc explainability analysis to justify the main features that contributed to the prediction output of the model. In addition, insights were given about the influence of each feature among the classes. 

Overall, we can conclude that the positive changes to a child’s life due to the first lockdown—the relationships among the family members, the time spent watching TV, and parental evaluation of the child’s mental health and the stress caused by COVID-19 restrictions—could play crucial role to the change in the mood state of the child. These results are aligned with the results of relevant studies found on the literature that incorporated pre-pandemic clinical samples or population-based cohorts of children at high risk for transition from subclinical to clinically significant levels of psychopathology [72,73,74]. Moreover, the finding that that most of the children and youths managed to maintain stable mood (63.04%: 469 out of 744) or even have positive mood change (14.25%: 106 out of 744) may be related to the concept of resilience. This is aligned to the psychological approach and perspectives on resilience in children and youth [68,69,70] and specifically on COVID-19 [71]. Specifically, these children seem to maintain their capacity for resilience, even under these difficult restrictive conditions. People may experience conditions of loss or high anxiety, but these may have little effect on their mental health, and positive aspects may even be experienced [75]. In a recent meta-analysis conducted by Prati and Mancini (2021), which also includes studies of children and adolescents, the psychological impact of COVID-19 lockdowns was small in magnitude, highlighting that most people are psychologically resilient to their effects [76]. There can be a positive adjustment of children after an acute life event, and the factors that contribute to it are both intra-individual and contextual factors (e.g., supportive relations) [77], as well as relationships with parents or the school’s ability to respond to the emergency [78]. Also, it seems that stability in functional routines is a key factor in managing stressful events. In accordance with this are the results of Giuntella et al. (2020), who found that disruptions in physical activity, sleep, and screen time among young adults at the onset of the pandemic are more closely linked to depression during the pandemic [79]. The results of the present study may be used to inform policy makers and clinicians in order to be prepared for similar crises or subsequent restriction periods (e.g., guidance for parents attending CAMHS).

The main limitations of this work that should be taken into account are the unexpected end of therapies by some children, and the fact that parents answered the questionnaires on the behalf of their children considering different time periods. Moreover, the large diversity of clinical diagnoses in combination with the small number of children falling into separate specifically defined diagnostic codes imposed the necessity to use broader diagnostic categories, and therefore to not succeed in observing the relation between the impact of COVID-19-related restrictions to children and diagnostic criteria from a specific disorder (e.g., ADHD). Future work includes a within-subject analysis of the data from the longitudinal study of the first and second lockdowns. It remains to be seen whether the second prolonged lockdown (six months) had a greater impact on the clustering of the population.

## Figures and Tables

**Table 1:** Lockdown policies implemented worldwide adapted from [3].

Type of Measures	Measures	Explanation
International Measures	Curfew	The effective date when a country announced a restriction on the movement of individuals within a given time of the day
	State of emergency	The effective date when a country announced a state of emergency
	Within-country regional lockdown	The effective date when a region within a country announced a total lockdown
	Partial selective lockdown	The earliest effective date for the partial restriction of the movement of people, i.e. school closures or limitations regarding the number of gathered people allowed
External measures	Selective international border closures	The earliest effective date when a country decided to close its borders with a region or country that has been significantly affected by COVID-19
	Selective border closures	The earliest effective date following the selective international border closure, when a country closed its border to individuals from one or multiple other countries that have been significantly affected by COVID-19
	International lockdown	The effective date when a country banned all flights, rail, and automotive movements internationally

**Table 2:** Summarization of studies related to the first COVID-19 outbreak, including children and young adults.

Study	Country	Population	Target	Method
[2]	China	8079 Chinese students aged 12–18	To identify correlations between sociodemographic features and mental health problems in Chinese adolescents during the outbreak of COVID-19	Multivariable logistic regression analysis
[4]	China	668 Chinese children aged 7–15	To identify the main factors that contribute to the education and the mental health of Chinese children during COVID-19	Multiple logistic regression analysis
[25]	China	584 youths	To study the effects of COVID-19 on youth mental health	Univariate analysis and univariate logistic regression
[14]	China	Two cross-sectional studies of 9554 and 3886 participants	To evaluate the factors that contribute to depression and anxiety among Chinese adolescents during COVID-19	Multivariable logistic regression analyses
[19]	China	1,199,320 school-aged children and adolescents	To assess the prevalence and the risk factors associated with self-reported psychological distress	Multivariate logistic regression
[21]	China	11,835 Chinese adolescents and young adults (12–29 years)	To identify sleeping problems during COVID-19	Binomial logistic regression analysis
[28]	China	2009 Chinese undergraduate students	To predict anxiety and insomnia during COVID-19	XGBoost model
[26]	China	746,217 Chinese university students	To examine variables associated with mental health problems during COVID-19	Univariate and hierarchical logistic regression analyses
[16]	China	89,588 Chinese university students	To identify the risk factors for anxiety symptoms during COVID-19	Multivariate logistic regression models
[17]	China	933 medical students	To evaluate the impact of COVID-19 on anxiety	Multivariate logistic regression
[18]	France	69,054 French university students	To study mental health issues due to COVID-19	Multivariate logistic regression
[15]	France	3671 participants	To identify the risk factors for depression during the COVID-19 pandemic	Multivariate logistic regression
[22]	Bangladesh	476 university students	To identify the risk factors for depression due to COVID-19	Binary logistic regression
[23]	Bangladesh	384 parents with at least one child aged 5–15	To identify mental health disturbances during COVID-19	Binary logistic regression
[20]	Canada	1013 children and adolescents aged 6–18, with or without pre-existing diagnoses	To evaluate the effects on mental health during COVID-19	Multinomial logistic regression
[24]	Brazil	157 girls and 132 boys aged 6–12	To examine the prevalence of anxiety during COVID-19	Logistic regression
[13]	Spain	523 adolescents (13–17 years)	To examine the association between sociodemographic factors and COVID-19-related variables and their effect on depression, anxiety, and stress	Multivariable logistic regression
[27]	Australia	Parents of 213 children and adolescents aged 5–17 who have been diagnosed with ADHD	To identify the impact of COVID-19 restrictions	Adjusted logistic regression analyses
[19]	China	478 college students after school reopening	To examine the psychological impact of COVID-19	Multivariate logistic regression
[30]	Belgium	2008 young people aged 16–25	To examine mental distress and its contributing factors	Bivariate and multivariable logistic regression analyses
[29]	Cross-sectional study	2787 participants aged 18–85	To identify predictors of psychological distress during COVID-19	Random forest machine learning algorithm and regression trees
[10]	Florida, USA	280 school-aged children	To examine mental health during COVID-19	Bivariate analysis and logistic and multinomial logistic regression models

**Table 3:** Sociodemographic characteristics of the dataset.

Sociodemographic Characteristics	Population (%)
Age, Mean ± Standard Deviation	10.7 ± 4.1
Sex Male Female Not willing to answer	466 (62.63%) 273 (36.7%) 5 (0.67%)
Participant parent Mother Father Other (grandparents, uncle/aunt, foster parents, other)	588 (79.7%) 142 (19.2%) 8 (1.1%)
Parent’s ethnicity Greek Other	725 (98.2%) 13 (1.8%)
Health insurance type National/Military Private Other None	650 (87.7%) 63 (8.7%) 9 (1.3%) 16 (2.3%)
Residential area City Suburbs of a city Town/village Rural area Island	382 (51.8%) 200 (27.1%) 131 (17.7%) 10 (1.4%) 15 (2.0%)
Reporting parent’s educational level Compulsory 9 years’ education Senior high school Institute of Vocational Training Technical College or University degree Postgraduate degree (M.Sc./PhD)	26 (3.5%) 146 (19.8%) 118 (16.0%) 280 (37.9%) 168 (22.8%)
Second parent’s educational level Compulsory 9 years’ education Senior high school Institute of Vocational Training Technical College or University degree Postgraduate degree (M.Sc., PhD)	80 (10.8%) 221 (29.9%) 105 (14.3%) 211 (28.6%) 121 (16.4%)
Essential worker (yes): healthcare, delivery worker, store worker, security, building maintenance	321 (43.5%)
Worker in a facility treating COVID-19 (yes)	105 (14.2%)
Job loss during the pandemic (yes)	38 (5.1%)
Limited ability to earn money (yes)	81 (10.9%)

**Table 4:** Dataset description.

Category	Features	Description
Demographics	age_group	Age group of child
	gender_child	Gender of child
	parent_area_live	Area of residence
	gender_parent	Gender of the parent or guardian
	parenteducation	Education level of parent or guardian
	school_child	School enrolment and attendance
	2w_essential_worker	Whether any adults living with the child are essential workers (health care, delivery services, pharmacies, law enforcement and security, store worker, cleaning services, other)
Social life	3m_outdoors	Days per week the child spent outside the house (parks, outdoor spaces) in 3 months and the past 2 weeks, respectively
	2w_outdoors	
	2w_time_outside	Amount of time per week the child spent/dedicated out of the house (e.g., shopping, parks, etc.)
	2w_event_cancellat	How difficult the cancellation of important events in the child’s life (graduation, vacation, Easter recess) was for him/her
	2w_recommendations	Difficulty following recommendations regarding social distancing
	2w_contact_changed	Change in the child’s contact with people outside home relatives compared to before the coronavirus/COVID-19 crisis
	2w_relationships_friends	Change in the quality of the child’s relationships with his/her friends
	3m_soc_media	Time spent using social media (e.g Facetime, Facebook, Instagram, Snapchat, Twitter, Tiktok) for 3 months and the past 2 weeks, respectively
	2w_soc_media	
Personal life	2w_positive	Positive changes in the child’s life due to the coronavirus/COVID-19 crisis
Family life	Family_impact_any	If any event that affected the family occurred due to COVID-19
	2w_financial_recod	Financial problems faced by the family due to the coronavirus/COVID-19 crisis
	2w_relationships_family	Changes in the quality of relationships between the child and members of his/her family
	2w_family_events_lost_job	Whether either of the following have happened to the child’s family members because of coronavirus/COVID-19: loss of job, loss of earnings
	2w_family_events_loss_earnings	
Daily activities	3m_exercise	Days per week the child engaged in exercise (e.g., increased heart rate, breathing) for at least 30 min, for 3 months and the past 2 weeks, respectively
	2w_exercise	
	2w_video_games	Time spent playing video games, for 3 months and the past 2 weeks, respectively
	3m_video_games	
	3m_tv	Time spent watching TV or digital means (e.g., Netflix, Youtube, or web surfing) for 3 months and the past 2 weeks, respectively
	2w_tv	
	2w_reading	How frequently the child asked questions, read, or talked about coronavirus/COVID-19
Health concerns	2w_worry_self_infected	Child’s worry about becoming infected
	2w_worry_family_inf	Child’s worry about family members or friends becoming infected
	2w_worry_phys_healt	Worry that physical health will be affected by coronavirus/COVID-19
	2w_worry_ment_health	Worry that the child’s mental/emotional health will be affected by coronavirus/COVID-19
Behavioral effects	2w_stress_restrict	Stress caused by the curfew
	2w_stress_family	Stress caused to the child by changes in family contacts
	2w_worry_food_reco	Worry about food in the family running out due to loss of income
	2w_stress_social	Stress caused to the child by changes to his/her social contacts
	2w_living_stability	Child’s concern about the stability of the family’s living situation
	2w_hopeful_end	How hopeful the child is that the coronavirus/COVID-19 crisis will end
Sleeping habits	3m_sleep_hours	Average sleep duration on weekdays, for 3 months and the past 2 weeks, respectively
	2w_sleep_hours_rec	
	3m_sleep_time	Sleep schedule on weekdays, for 3 months and the past 2 weeks, respectively
	2w_sleep_time_reco	
	3m_sleep_hours_weeke	Average sleep duration on weekends, for 3 months and the past 2 weeks, respectively
	2w_sleep_hours_wee	
	3m_sleep_time_weeken	Sleep schedule on weekends, for 3 months and the past 2 weeks, respectively
	2w_sleep_time_week	
Medical diagnosis/rehabilitation	2w_child_health_evaluation	Parental evaluation of the child’s overall physical health before the coronavirus/COVID-19 crisis
	2w_mental_health_eval	Parental evaluation of the child’s overall mental/emotional health before the coronavirus/COVID-19 crisis
	diagnosis_1_group	Diagnosis defined by the medical expert
	Diagnosis_FINAL_groups	Final diagnostic category defined by the medical expert
	2w_symptoms_tot	Symptoms the child had
	2w_all_exposure_tot	Child exposed to someone likely to have coronavirus/COVID-19
	2w_support_activit	Supports which were in place for the child and have been disrupted
	2w_family_diagnosis	Whether any members of the child’s family have been diagnosed with COVID-19
	2w_family_events_ho	Whether any of the following have happened to the child’s family members because of Coronavirus/COVID-19: Hospitalization, self-quarantine, death, physical illness; and total number of the above family events
	2w_family_events_qu	
	2w_family_events_di	
	2w_family_events_il	
	2w_family_events_to	
Mood state	3m_general_worry 2w_general_worry	How worried the child generally was, 3 months ago and over the past 2 weeks, respectively
	3m_sadness 2w_sadness	How happy versus sad the child was, 3 months ago and over the past 2 weeks, respectively
	3m_anxiety 2w_anxiety	How relaxed versus anxious the child was, 3 months ago and over the past 2 weeks, respectively
	3m_restlessness 2w_restlessness	How fidgety or restless the child was, 3 months ago and over the past 2 weeks, respectively
	3m_anhedonia 2w_anhedonia	Ability of the child to enjoy his/her usual activities, 3 months ago and over the past 2 weeks, respectively
	3m_loneliness 2w_loneliness	How lonely the child was, 3 months ago and over the past 2 weeks, respectively
	3m_irritability 2w_irritability	How irritable or easily angered the child was, 3 months ago and over the past 2 weeks, respectively
	3m_concentration 2w_concentration	How well the child was able to concentrate or focus, 3 months ago and over the past 2 weeks, respectively
	3m_tiredness 2w_tiredness	How fatigued or tired the child was, 3 months ago and over the past 2 weeks, respectively
	3m_rumination 2w_rumination	How often the child was expressing negative thoughts, 3 months ago and over the past 2 weeks, respectively

**Table 5:** Summarization of classifiers.

Classifier	Description
Random Forest	An extended version of a decision tree that predicts the future instances with multiple classifiers, rather than a single classifier, to reach an accurate and correct prediction. RF constructs a large number of decision trees. Each decision tree denotes a class prediction, and the class with the most votes represents the model’s prediction [56].
Multi-Layer Perceptron	MLP belongs in the category of Artificial Neural Networks (ANN) and it is the most common neural network. MLP is based on a supervised training procedure to generate a nonlinear model for prediction. It consists of layers, such as the input layer, output layer, and hidden layers. Thus, MLP is a layered feedforward neural network where the information is transferred unidirectionally from the input layer to the output layer through the hidden layers [29].
Extreme Gradient Boosting	XG Boost is an extendible and cutting-edge application of gradient-boosting machines. Gradient boosting is an algorithm in which new models are created to predict the residuals of prior models, and then added together to make the final prediction. It uses a gradient descent algorithm to minimize the loss when adding new models [57].
Logistic Regression	A mathematical model that describes the relationship of data to a dichotomous dependent variable. The model is based on the logistic function, $f(x)\;=\;\frac{1}{1+e^{-x}}$ *where x* ∈ (−∞, +∞) and 0 ≤ *f*(*x*) ≤ 1. Thus, regardless the value of x the model is designed to describe the data with a probability in the range of 0 and 1 in a A-shaped graph [58].
Support Vector Machine	SVM is a supervised learning model based on the statistical learning framework, called VC theory. SVM targets to create a decision boundary, the hyperplane, between two classes, which enables the prediction of labels from one or more feature vectors, such that the distance between the closest points of each class, called support vectors, and the hyperplane to be maximized [59].
K-Nearest Neighbor	KNN is a non-parametric classification method that tries to classify an unknown sample based on the known classification of its neighbors [60].
Decision Trees	DTs are sequential models, which logically combine a sequence of simple tests. Each test compares a numeric attribute against a threshold value or a nominal attribute against a set of possible values [61].

**Table 6:** Parameter settings for clustering methods.

Clustering Method	Parameter Settings
Mini Batch K-Means	3 classes
Spectral Clustering	3 classes, arpack eigen solver, nearest_neighbors affinity
Ward’s Hierarchical Agglomerative Clustering	3 classes, ward linkage, symmetric connectivity
Average Linkage	3 classes, average linkage, cityblock affinity, symmetric connectivity
Birch	3 classes
Jenks	3 classes, include lowest value

**Table 7:** Hyper parameter settings for tuning the ML algorithms.

Classification Model	Hyper Parameters Tuning
Random Forest	n_estimators = [int(x) for x in np.linspace(start = 10, stop = 500, num = 10)]; max_features = [‘auto’, ‘sqrt’]; max_depth = [int(x) for x in np.linspace(3, 10, num = 1)]; min_samples_split = [3, 4, 5, 6, 7, 10]; min_samples_leaf = [1, 2, 4]; bootstrap = [True, False].
Multi-Layer Perceptron	hidden_layer_sizes = [(2, 5, 10), (5, 10, 20), (10, 20, 50)]; activation = [‘tanh’, ‘relu’]; solver = [‘sgd’, ‘adam’]; alpha = [0.0001, 0.05]; learning_rate = [‘constant’, ‘adaptive’]
XG Boost	max_depth = [2, 3, 4, 5, 6, 7, 8]; min_child_weight = [1, 2, 3, 4, 5, 6]; gamma = [0, 0.4, 0.5, 0.6]
Logistic Regression	C = [0.001, 0.01, 0.1, 1, 2, 3, 4, 5, 6, 7, 8, 9, 10]; warm_star = [True, False]; multi_class = [‘ovr’, ‘multinomial’]; solver = [‘newton-cg’, ‘lbfgs’, ‘sag’, ‘saga’]
Support Vector Machine	C = [0.001, 0.01, 0.1, 1, 2, 3, 4, 5, 6, 7, 8, 9, 10]; kernel = [‘linear’, ‘sigmoid’, ‘rbf’, ‘poly’]
K-Nearest Neighbor	n_neighbors = [5, 7, 9, 12, 14, 15, 16, 17]; leaf_size = [1, 2, 3, 5]; weights = [‘uniform’, ‘distance’]; algorithm = [‘auto’, ‘ball_tree’, ‘kd_tree’, ‘brute’]
Decision Trees	max_features = [‘auto’, ‘sqrt’, ‘log2’]; min_samples_split = [2, 3, 4, 5, 6, 7, 8, 10, 12, 15]; min_samples_leaf = [1, 2, 3, 4, 5, 6, 7, 8, 10]

**Table 8:** Clustering results.

Clustering Methods	Cluster Information	Clusters		
		Cluster 0	Cluster 1	Cluster 2
Mini Batch K-Means	Set	[−24, −4]	[−3, 4]	[5, 25]
	Number of elements	144	468	132
Spectral Clustering	Set	Unable to create continuous sets		
	Number of elements	485	230	29
Ward	Set	[−24, −7]	[−6, 1]	[2, 25]
	Number of elements	66	418	260
Average Linkage	Set	[−24, −7]	[−6, 4]	[5, 25]
	Number of elements	66	546	132
Birch	Set	[−24, −6]	[−5, 8]	[9, 25]
	Number of elements	80	608	56
Jenks	Set	[−24, −5]	[−4, 3]	[4, 25]
	Number of elements	106	469	169

**Table 9:** Evaluation of clustering methods. The best evaluation score is shown in bold.

Clustering Method	Evaluation Method			Cumulative Normalized Score
	Silhouette Coefficient	Calinski–Harabasz Index	Davies–Bouldin Index	
Mini Batch K-Means	0.55	1106.78	0.60	2.94
Spectral Clustering	0.12	24.95	14.79	0.00
Ward	0.54	989.18	0.58	2.80
Average Linkage	0.57	1048.06	0.52	2.94
Birch	0.55	784.60	0.49	2.64
Jenks	0.56	1112.73	0.58	2.96

**Table 10:** Results from feature selection with the categories of the 40 first features.

Features	Category	Features	Category
1st feature	Social life	21st feature	Daily activities
2nd feature	Behavioral effects	22nd feature	Behavioral effects
3rd feature	Medical diagnosis/rehabilitation	23rd feature	Behavioral effects
4th feature	Social life	24th feature	Social life
5th feature	Personal life	25th feature	Daily activities
6th feature	Medical diagnosis/rehabilitation	26th feature	Daily activities
7th feature	Demographics	27th feature	Medical diagnosis/rehabilitation
8th feature	Family life	28th feature	Demographics
9th feature	Family life	29th feature	Behavioral effects
10th feature	Social life	30th feature	Health concerns
11th feature	Social life	31st feature	Sleeping habits
12th feature	Daily activities	32nd feature	Social life
13th feature	Daily activities	33rd feature	Demographics
14th feature	Health concerns	34th feature	Social life
15th feature	Daily activities	35th feature	Medical diagnosis/rehabilitation
16th feature	Health concerns	36th feature	Social life
17th feature	Demographics	37th feature	Sleeping habits
18th feature	Behavioral effects	38th feature	Sleeping habits
19th feature	Social life	39th feature	Sleeping habits
20th feature	Health concerns	40th feature	Demographics

**Table 11:** The maximum accuracy achieved from the classification models. The best performance is shown in bold.

Models	Maximum Accuracy (%)	Number of Features for Maximum Accuracy
Random Forest	66.60	44
MLP	57.73	58
XG Boost	**69.47**	**40**
Logistic Regression	55.44	50
SVM	64.05	49
KNN	51.28	3
Decision Trees	53.23	5

**Table 12:** Results after XG Boost classifier calibration with Isotonic Regression and Platt’s methods. The best scores are shown in bold.

Models	Log-Loss	Accuracy (%)
XG Boost	1.195	69.47
XG Boost + Isotonic	0.513	72.03
XG Boost + Platt	**0.489**	**76.52**

## Data Availability

The gathered data are strictly for use within the research project and are not publicly available for the moment.
