# Correction: Ntakolia et al. An Explainable Machine Learning Approach for COVID-19’s Impact on Mood States of Children and Adolescents during the First Lockdown in Greece. *Healthcare* 2022, *10*, 149

**DOI:** 10.3390/healthcare10040657

**Published:** 2022-03-31

**Authors:** Charis Ntakolia, Dimitrios Priftis, Mariana Charakopoulou-Travlou, Ioanna Rannou, Konstantina Magklara, Ioanna Giannopoulou, Konstantinos Kotsis, Aspasia Serdari, Emmanouil Tsalamanios, Aliki Grigoriadou, Konstantina Ladopoulou, Iouliani Koullourou, Neda Sadeghi, Georgia O’Callaghan, Eleni Lazaratou

**Affiliations:** 1 University Mental Health Research Institute, 11527 Athens, Greece; icedale@gmail.com (D.P.); mariana.har.travlos@gmail.com (M.C.-T.); ioannarannou@gmail.com (I.R.); 2 First Psychiatric Department, Eginition Hospital, National and Kapodistrian University of Athens, 11528 Athens, Greece; nadia.magklara@gmail.com (K.M.); elazar@med.uoa.gr (E.L.); 3 Second Psychiatric Department, ‘Attikon’ University Hospital, National and Kapodistrian University of Athens, 12462 Athens, Greece; igioannag@gmail.com; 4 Department of Psychiatry, Faculty of Medicine, School of Health Sciences, University of Ioannina, 45110 Ioannina, Greece; konkotsis@gmail.com; 5 Department of Child and Adolescent Psychiatry, Medical School, Democritus University of Thrace, University Hospital of Alexandroupolis, 68100 Alexandroupolis, Greece; aserntar@med.duth.gr; 6 Department of Child and Adolescent Psychiatry, Division of Psychiatry, ‘Asklepieion Voulas’ General Hospital, 16673 Attica, Greece; emtsalamanios@hotmail.com; 7 Hellenic Centre for Mental Health and Research, 10683 Athens, Greece; alikigrigoriadou@gmail.com; 8 Athens Child and Adolescent Mental Health Centre, General Children’s Hospital ‘Pan. & Aglaia Kyriakou’, 11527 Athens, Greece; kladopou@gmail.com; 9 Mental Health Center, General Hospital ‘G. Hatzikosta’, 45445 Ioannina, Greece; jkoullourou@gmail.com; 10 Section of Clinical and Computational Psychiatry, National Institute of Mental Health, National Institutes of Health, Bethesda, MD 20892, USA; neda.sadeghi@nih.gov (N.S.); georgiaocallaghan@gmail.com (G.O.)

## Exclusion of an Author

**Argyris Stringaris** was initially included as an author in the original publication [1]. However, due to his personal decision, we have excluded him from the authors and we added his contribution to the acknowledgments section. The corrected Author Contributions and Acknowledgments are shown below.

**Author Contributions:** Conceptualization, C.N.; methodology, C.N.; software, C.N.; validation, C.N.; formal analysis, C.N., I.R., I.G., A.S. and E.L.; data curation, C.N., D.P., K.M., I.G., K.K., A.S., E.T., A.G., K.L., I.K., N.S. and G.O.; writing—original draft preparation, C.N., D.P., I.R., A.S., E.L. and M.C.-T.; writing—review and editing, C.N., I.R., I.G. and M.C.-T.; visualization, C.N.; supervision, E.L.; project administration, E.L. All authors have read and agreed to the published version of the manuscript.

**Acknowledgments:** The authors would like to thank all the respondents to this study who took the time to complete the questionnaire. We would like to thank Argyris Stringaris for his contributions to coordinating sample collection and discussions regarding the clinical aspects of the paper. We would also like to thank the Hellenic COVID-19 imPact survEy (HOPE) Consortium for their contribution during the data collection process: Lagakou E., First Psychiatric Department, Eginition Hospital, National and Kapodistrian University of Athens, Athens, Greece; elagakou@gmail.com. Mamaki, E., Mental Health Center, General Hospital “G. Hatzikosta”, Ioannina, Greece; g.vottis@yahoo.gr. Neou, E., Hellenic Centre for Mental Health and Research, Athens, Greece; evaneou@gmail.com. Polaki, O., Community Mental Health Center for Children and Adolescents in N.Smyrni, Division of Psychiatry, “Asklepieion Voulas’ General Hospital, Attica, Greece; olympiapolaki@yahoo.gr. Priftis D., University Mental Health Research Institute; icedale@gmail.com. Triantafyllou, G., Second Psychiatric Department, “Attikon” University Hospital, National and Kapodistrian University of Athens, Athens, Greece; g_triantafillou@yahoo.gr. Valvi E., Athens Child and Adolescent Mental Health Centre, General Children’s Hospital “Pan. & Aglaia Kyriakou”, Athens, Greece. Vassara, V., Community Mental Health Center for Children and Adolescents, Department of Psychiatry, University Hospital of Ioannina, Ioannina, Greece; vasilikivassara@gmail.com.

The authors apologize for any inconvenience caused and state that the scientific conclusions are unaffected. The original publication has also been updated.
